# Bacterial Sigma Factors and Anti-Sigma Factors: Structure, Function and Distribution

**DOI:** 10.3390/biom5031245

**Published:** 2015-06-26

**Authors:** Mark S. Paget

**Affiliations:** School of Life Sciences, University of Sussex, Falmer, Brighton BN1 9QG, UK; E-Mail: m.paget@sussex.ac.uk; Tel.: +44-1273-877-764

**Keywords:** RNA polymerase, transcription, anti-sigma, sigma, partner-switching, RIP, signal transduction, stress, extracytoplasmic

## Abstract

Sigma factors are multi-domain subunits of bacterial RNA polymerase (RNAP) that play critical roles in transcription initiation, including the recognition and opening of promoters as well as the initial steps in RNA synthesis. This review focuses on the structure and function of the major sigma-70 class that includes the housekeeping sigma factor (Group 1) that directs the bulk of transcription during active growth, and structurally-related alternative sigma factors (Groups 2–4) that control a wide variety of adaptive responses such as morphological development and the management of stress. A recurring theme in sigma factor control is their sequestration by anti-sigma factors that occlude their RNAP-binding determinants. Sigma factors are then released through a wide variety of mechanisms, often involving branched signal transduction pathways that allow the integration of distinct signals. Three major strategies for sigma release are discussed: regulated proteolysis, partner-switching, and direct sensing by the anti-sigma factor.

## 1. Introduction

In bacteria, the initiation of transcription at promoters requires a dissociable specificity subunit of RNA polymerase (RNAP) called sigma (σ) that binds to the core (subunits ββ'α_2_ω) to form the “holoenzyme”. σ factors play distinct roles at different stages of initiation including the direct recognition of promoter elements to form an initial “closed” complex (RP_c_), stabilisation of the “open” complex (RP_o_) in which DNA around the transcription start site is melted, interaction with transcription activators, the stimulation of the early steps in RNA synthesis, and can influence promoter escape (reviewed in [1]). They can be classified into two distinct families based on their homology to two σ factors in *Escherichia coli*: the primary σ factor σ^70^ that is responsible for the bulk of transcription during growth; and the structurally unrelated σ^54^ (or σ^N^) that directs transcription in response to environmental signals, and requires the input of enhancer proteins and ATP hydrolysis to drive DNA melting (reviewed in [2]). This review focuses solely on the σ^70^ family. σ^70^ family members consist of up to four structurally conserved domains connected by flexible linkers that bind across one face of RNAP. In the holoenzyme, the DNA binding determinants of σ are exposed, allowing their interaction with promoter elements centered −35 bp and −10 bp upstream from the transcription start site. When RNAP escapes the promoter to enter the elongation phase, σ dissociates in stochastic fashion and only rebinds RNAP following termination, although there is evidence σ can associate with RNAP at transcription pause sites [3,4]. This “σ cycle” enables a regulatory strategy that involves the controlled production of alternative σ factors that can redirect RNAP to distinct promoters [5]. Following early discoveries of alternative σ factors involved in stress responses and sporulation in *Bacillus subtilis* [6,7] and the heat-shock response in *E. coli* [8], it became clear that the deployment of alternative σ factors to co-ordinately induce gene expression is widespread in bacteria. Indeed, large scale genome sequencing has revealed a plethora of σ factors in some organisms with, for example, 109 encoded by the Gram-negative myxobacterium *Sorangium cellulosum* [9]. The σ^70^ family has been classified into four major phylogenetically and structurally distinct groups with Group 1 consisting of primary σ factors and Groups 2–4 comprised of alternative σ factors with specialised functions. The expression and activity of alternative σ factors can be controlled at many levels, but particularly prevalent is their post-translational control by anti-σ factors that prevent their interaction with RNAP. Anti-σ factors use diverse mechanisms to release the σ in response to specific stimuli, often involving mechanisms that transduce extracellular signals to the cytoplasm. In this review, the structure and function of the σ^70^ family is considered, along with selected mechanisms for their control by anti-σ factors.

## 2. Structural Organisation of σ^70^ and Other Group 1 σ Factors

**Figure 1:**
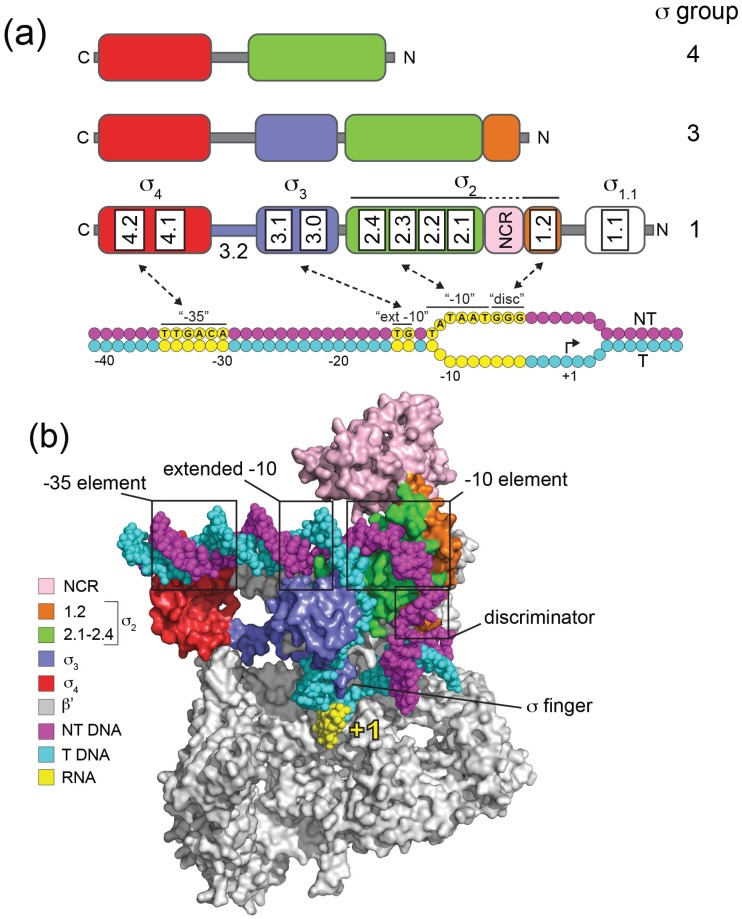
Domain organization, promoter recognition and structural organization of the σ^70^ family. (**a**) The domain organization of σ factors from Groups 1, 3 and 4 are illustrated above σ^70^ (Group 1) consensus *E. coli* promoter DNA. Structural domains are colored: σ_1.1_, white; σ_2_, green/orange; σ_3_, blue; σ_4_, red. Within each domain, conserved σ regions are indicated for Group 1 σs. Non-template (NT) strand DNA is colored magenta and template (T) strand cyan, with key consensus promoter elements contacted by σ indicated in yellow: “−35”, −35 element; “ext −10”, extended −10 element; “−10”, −10 element; “disc”, discriminator. Transcription initiates at +1. Note that σ_2_ is colored green and orange to distinguish σ regions 2.1–2.4 and 1.2. The nonconserved region (NCR; pink) located between 1.2 and 2.1 (pink) is variable in size and structure among Group 1 σ factors. (**b**) Organization of *E. coli* σ^70^ in an RNA polymerase transcription initiation complex. The model was based on the crystal structure of an *E. coli* transcription initiation complex (PDB: 4YLN) [22]. σ^70^ domains (surface representation) and promoter DNA (spheres) are colored as in (a), as indicated in the panel. For clarity the β, 2α and ω subunits of RNA polymerase are omitted. The model indicates the location of the σ finger and its close proximity to nascent RNA (4 nt, yellow) and template strand DNA.

Groups 1 to 4 in the σ^70^ family differ by the presence and absence of four conserved regions (σ Regions σR1.1, σR1.2–2.4, σR3.0–3.2, σR4.1–4.2; [10,11]) that reflect four helical structured domains (σ_1.1_, σ_2_, σ_3_, σ_4_) that have been determined by studies on isolated fragments [12,13] or in the context of holoenzyme [14,15,16] (Figure 1).

**Figure 2:**
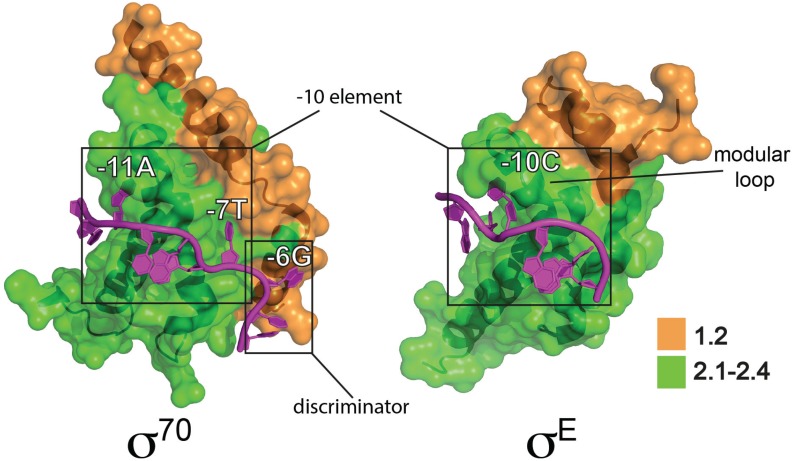
Interactions of *E. coli* σ^70^ and σ^E^ with “flipped out” bases in non-template strand of −10 regions. The σ^70^-DNA model is a partial representation of a crystal structure of an *E. coli* σ^70^ transcription initiation complex (PDB: 4YLN) [22] showing the σ^70^_2_ domain interactions with the non-template strand (C_-13_T_-12_A_-11_T_-10_A_-9_A_-8_T_-7_G_-6_T_-5_G_-4_) Flipped out bases in the −10 element (−11A and −7T) and the discriminator element (−6G) are indicated. Note that DNA upstream of −11A is double stranded, with the template strand not shown for clarity. The σ^E^-DNA model is based on a crystal structure of the *E. coli* σ^E^_2_ domain bound to a non-template strand oligonucleotide (PDB: 4LUP) [23] based on the σ^E^ consensus −10 sequence (T_-13_G_-12_T_-11_C_-10_A_-9_A_-8_A_-7_). The modular loop that interacts with the flipped out −10C is indicated. σ_2_ domains are illustrated in surface representation with conserved regions colored as indicated and the σ^70^ NCR not shown.

Domains σ_2_, σ_3_ and σ_4_ each interact with specific promoter elements and with RNAP (Figure 1). Domain σ_2_ (σR1.2–2.4) is the most conserved, and forms part of an extensive interface with RNAP primarily involving an α-helix comprising σR2.2 and the β' coiled-coil [14]. During the process of DNA melting σ_2_ makes base-specific interactions with the single-stranded non-template DNA of the −10 element (σR2.3–2.4) thereby capturing the DNA and stabilizing RP_o_ (Figure 1). The *E. coli* σ^70^ −10 consensus sequence (T_-12_ATAAT_-7_) is particularly highly conserved at A_-11_ and T_-7_, since these bases are flipped out of the base stack and buried in complementary σ_2_ pockets in RP_o_ [17,18] (Figure 2). In Group 1 and Group 2 σ factors σR1.2, consisting of two α helices oriented 90° to one another, interacts with the non-template strand “discriminator” element, which has an optimum sequence 5'-GGG-3' and is located immediately downstream of the −10 element [18] (Figure 1 and Figure 2). The discriminator was originally identified because its natural absence from ribosomal RNA promoters confers instability to open complexes, which facilitates regulation by the stringent factor ppGpp during nutrient stress [19,20,21].

Domain σ_3_ is a compact three-helix bundle that interacts with the major groove of duplex DNA just upstream from the −10 element [24] (Figure 1). Interactions with these “extended −10” elements (T_-15_G_-14_ in *E. coli*) can stabilize initiation complexes to such an extent that the otherwise crucial −35 element is not required. Domain σ_4_ (σR4.1–4.2) is comprised of four helices with the third and fourth forming a helix-turn-helix motif that binds to the −35 element (Figure 1). Domain σ_4_ forms the second largest interface with RNAP through its interaction with the β flap, and also acts as a contact point for transcriptional activators that bind DNA upstream of −35. A conserved linker (σR3.2) between σ_3_ and σ_4_ threads through the RNAP active site channel and occupies the RNA exit path, emerging from under the β flap (Figure 1b). Part of the linker, termed the “σ finger”, facilitates transcription initiation by interacting directly with the template strand, but needs to be displaced when >4 nt RNA have been synthesized [18] (Figure 1b). Domain σ_1.1_ (σR.1.1) is only found in Group 1 and promotes a compact form of free σ, occluding the DNA binding determinants, and thereby inhibiting its non-productive interaction with promoter DNA in the absence of core [13,25]. However, upon holoenzyme formation, the negatively-charged σ_1.1_ acts as a DNA mimic, occupying the RNAP active site channel at a position that will subsequently be occupied by duplex DNA in RP_o_ [16,26,27]. σ_1.1_ therefore needs to be displaced during RP_o_ formation, such that it acts as a “gatekeeper” that stimulates isomerisation at some promoters while inhibiting the process at others [28,29]. Some Group 1 σ factors additionally contain a non-conserved region (NCR) between σR1.2 and σR2.1 that is of variable length and composition and has been implicated in core binding and promoter escape in the case of *E. coli* σ^70^ [30] (Figure 1).

## 3. Structure and Function of Alternative σ Factors

Despite their large variation in size, from ~70 kDa for Group 1 to ~20 kDa for Group 4, all members of the σ^70^ family possess the σ_2_ and σ_4_ domains that include the major RNAP- and promoter-binding determinants (Figure 1a). However, alternative σ factors differ from Group 1 σ factors by the complete absence of σ_1.1_, the variable presence of σ_3_, promoter specificity, and in some aspects of initiation. The extent to which alternative σ factors are used varies enormously between bacteria, as does their range of functions, from sensing and responding to a wide variety of extracellular and intracellular signals, to providing regulatory output to morphological checkpoints.

### 3.1. Group 2 σ Factors

Group 2 σ factors are structurally closely related to Group 1, but lack σ_1.1_ and are non-essential. Where studied, Group 2 σs are usually involved in adaptation to stress including nutrient limitation and other stresses associated with stationary phase. Multiple Group 2 σs exist in some organisms including three with unknown role in *Streptomyces coelicolor* [31] and at least four encoded in cyanobacterial genomes. In *Synechocystis* 6803 these σ factors have been implicated in the response to a broad range of stresses including high temperature, light regulation and sugar metabolism [32]. For example, *Synechocystis* 6803 σ^E^ has a role in sugar metabolism and is required for light-activated heterotrophic growth [33]. In *Synechococcus elongatus* disruption of any of the four Group 2 σs alters circadian expression [34], and although it is not clear whether these are direct or pleiotropic effects, the expression of two of these (*rpoD5*/*rpoD6*) exhibited high-amplitude rhythms distinct from that of the principal σ [35]. The best-studied Group 2 σ is *E. coli* σ^S^ (also known as σ^38^) that is responsible for the general stress response and for survival during stationary phase, and is induced in response to a variety of environmental stresses [36]. Its expression is influenced by growth rate [37] and it positively influences the expression of nearly 500 genes under different stress conditions [38]. Not surprisingly, considering the extensive sequence conservation between σ^70^ and σ^S^ in the DNA binding determinants, promoters recognized by these σ factors are highly similar, although differences include a C nucleotide located at −13 in an extended −10 promoter element that is thought to interact with σ^S^_3_ [39].

### 3.2. Group 3 σ Factors

Members of this group are structurally and functionally diverse, but usually contain σ_2_, σ_3_ and σ_4_ domains. The −10 and −35 elements are varied among members and distinct from those recognised by Groups 1 and 2, although the presence of σ_3_ correlates with the recognition of extended −10 elements in some cases [40,41]. They fall into at least four phylogenetically-distinct subgroups that partially correlate with function: flagellum biosynthesis, heat shock response, general stress, and sporulation [11,42]. (1) The expression of “late” genes involved in flagellum biosynthesis is controlled by σ factors closely related to *E. coli* σ^28^ (FliA) in all motile Gram-negative and Gram-positive bacteria, making this the most widely distributed alternative σ factor. Indeed, the *B. subtilis* orthologue σ^D^ can complement an *E. coli fliA* mutant, highlighting conservation in promoter recognition as well as function [43]. Closely related paralogues are also found in non-motile bacteria including *Streptomyces* (σ^WhiG^) where it is required for sporulation [44] and the obligate intracellular parasite *Chlamydia trachomatis* where it is developmentally regulated [45]. (2) In α- and γ-proteobacteria, the response to the accumulation of unfolded proteins in the cytoplasm caused by heat shock and other stresses is controlled by σ factors related to *E. coli* σ^32^. (3) In Gram-positive bacteria, the general stress response is controlled by σ factors related to *B. subtilis* σ^B^. In *B. subtilis*, σ^B^ controls almost 200 genes in response to a wide range of challenges including environmental stressors such as ethanol, osmotic stress, and nitric oxide, as well as energy-related stresses such as ATP depletion [46,47]. Although most organisms have a single σ^B^-like protein, up to 9 paralogues are present in *Streptomyces* that are involved in both stress responses and development [48,49,50]. (4) Endospore formation in *B. subtilis* is driven by four related compartment-specific Group 3 σ factors (σ^F^, σ^E^, σ^G^, σ^K^). At an early stage of sporulation, asymmetric cell division forms a pre-spore compartment that is ultimately engulfed by the “mother cell”. The activation of σ^F^ in the pre-spore triggers a criss-cross signalling cascade that results in the sequential activation of σ^E^ (mother cell), σ^G^ (pre-spore), and σ^K^ (mother cell) [51].

### 3.3. Group 4 (ECF) σ Factors

Group 4 is also known as the ExtraCytoplasmic Function (ECF) group on account of the frequent role of members in sensing and responding to signals that are generated outside of the cell or in the cell membrane [52]. The ECF group is numerically by far the largest and most diverse at the primary sequence level, consisting of at least 43 major phylogenetically distinct sub-groups [53]. Biological roles include envelope stress response (e.g., *E. coli* σ^E^, *B. subtilis* σ^W^), iron transport (*E. coli* σ^FecI^, *Pseudomonas aeruginosa* σ^PvdS^), oxidative stress (*S. coelicolor* σ^R^ and *Rhodobacter sphaeroides* σ^E^), and the general stress response (e.g., *Methylobacterium extorquens* σ^EcfG1^). The number of ECF σ factors encoded by a genome can vary enormously from zero in obligate intracellular bacteria such as Chlamydiae to over 115 in the planktomycete *Gemmata obscuriglobus* [54]. Some ECF subgroups are widespread in bacterial phyla, indicating an ancient origin, while most tend to be phylum-specific. Indeed in their initial classification of 2700 ECF σ factors, Mascher and colleagues identified 24 minor groups and hundreds of proteins that could not be sub-classified [53]. Clearly, as genome sequencing technology is directed towards less studied phyla, many additional major and minor subgroups will be uncovered. The presence of multiple ECF σ factors raises the question of whether cross-talk occurs, such that some promoters are recognised by several σ factors, potentially allowing the integration of distinct signals towards a coordinated response. This is the case in *B. subtilis* where cell envelope stress response is coordinated by several ECF σ factors with partially overlapping regulons [55]. Nonetheless, a study that tested for orthogonality among the broad spectrum of ECF σ factors concluded that there is generally little cross-talk between different subclasses, ensuring insulated transcriptional responses [56].

ECF σ genes are often clustered with the genes they regulate when the regulons are small, although some, such as σ^R^ in *S. coelicolor*, target >100 genes [57,58]. ECF σ factors lack both σ_1.1_ and σ_3_, which makes them the most minimal σ factors (Figure 1a). They also lack the first helix of σR1.2 that is responsible for discriminator interactions in Group 1/2 σ factors [18,20] (Figure 2). While most are small, there are exceptions including members of the widely distributed ECF41 subfamily that have ~100 amino acid residue C-terminal extensions that are thought to modulate activity [59]. Promoters recognised by ECF σ factors include a −35 “AAC” motif in two-thirds of examples and more diverged sequences at −10 [53], suggesting that promoter specificity is derived primarily from interactions between σ_2_ and the −10 element (although −35 interactions are also important [56]). Consistent with this, key residues that interact with the −10 region are on a variable loop. The recent structure of *E. coli* σ^E^_2_ in complex with non-template −10 ssDNA (G_-12_TCAAA_-7_) revealed that, unlike the case for Group 1 σ factors, only a single base (C_-10_) was flipped out from the ssDNA base stack [23] (Figure 2). This nucleotide is wrapped by a helical hinge in σR2.3 (α3–loop L3–α4) with the loop becoming ordered upon C_-10_ binding. Remarkably, the replacement of this loop in σ^E^ with the equivalent regions from other ECF σ factors with altered −10 recognition switched promoter specificity. This suggests that the variable loop provides a modular interface that contributes to the diversity of −10 elements and therefore the expansion of this class of σ factors [23].

## 4. Inhibition of Alternative σ Factors by Anti-σ Factors

**Figure 3:**
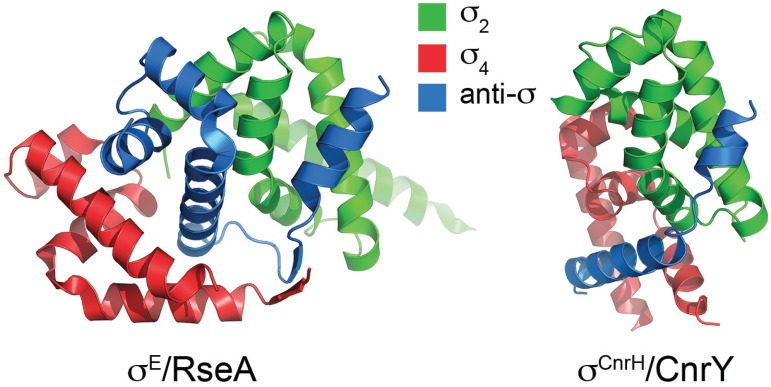
The inhibition of σ activity by anti-σ factors. A comparison between complexes of σ^E^-RseA (PDB: 1OR7-2) and a σ^CnrH^/CnrY/ (PDB: 4CXF). The σ_2_ and σ_4_ domains are colored green and red, respectively. The anti-sigma binding domains of RseA and CnrY are colored blue.

A variety of mechanisms control both the cellular concentration of alternative σs and their association with core RNAP. σ concentration might be controlled at the level of transcription, translation and protein turnover. Some “pro-σ” factors are activated by controlled proteolysis, involving the removal of an inhibitory extension, commonly located at the N-terminus (e.g., the activation of the pro-σ^E^ and pro-σ^K^ in the mother cell during sporulation in *B. subtilis*). However, particularly common among alternative σ factors is their control by specific anti-σ proteins that impede RNAP binding. The σ factor is then released in response to a signal that is perceived either by the anti-sigma factor itself or by additional components in more complex signal-relay type systems. Therefore, anti-σ factors are often modular, consisting of a σ-binding domain and a sensory/signalling domain that responds to a signal either within or outside of the cell. Unlike σ factors, anti-σ factors are poorly conserved at the primary sequence level. However, they are often co-transcribed with σ factor genes, which might help to ensure that stoichiometric levels are maintained. In general, anti-σ factors stabilise the σ in a form that is incompatible with RNAP binding by occluding the key RNAP binding determinants through bipartite interactions with σ_2_ and σ_4_ (Figure 3). Our understanding of the mechanism of σ factor inhibition has benefited from structures of several σ /anti-σ complexes [60] and some are discussed here to illustrate themes.

### 4.1. Anti-σ Factors that Insert between σ_2_ and σ_4_

Up to one third of ECF σ factors are controlled by anti-σs that share an N-terminal anti-sigma domain (ASD) structural motif [61]. The structure of *E. coli* σ^E^ in complex with the N-terminal cytoplasmic domain of its anti-σ RseA revealed that the first three RseA helices (ASD-α1-3) form a three-helix bundle that is sandwiched between the σ_2_ and σ_4_ domains and occupies a groove in σ_4_ that occludes the β-flap-tip-helix binding determinant (Figure 3) [62]. The fourth helix, ASD-α4, interacts with σR2.2 thereby blocking β' coiled-coil interactions. Despite lacking sequence similarity, ChrR, a two-domain anti-σ factor that controls the *R*. *sphaeroides* σ^E^-dependent response to singlet oxygen, has a highly similar ASD three-helix bundle buried between the cognate σ_2_ and σ_4_ domains (albeit with different juxtaposition to σ^E^/RseA) with ASD-α4 also blocking β' coiled-coil interactions [61]. Interestingly, whereas the RseA ASD fold is stabilized by extensive hydrophobic interactions, ChrR uses a structural zinc that is coordinated by a common ZASD (zinc binding anti-sigma domain) motif that was initially recognized in the *S. coelicolor* redox sensing anti-σ^R^ factor RsrA [61,63]. The ZASD motif includes a HisXXXCysXXCys sequence that includes three of the four zinc ligands, with the fourth ligand being either cysteine or histidine.

### 4.2. Anti-σ Factors that Wrap around σ_2_ and σ_4_

An alternative mechanism is to stabilize the naturally compact structure that free σ factors are thought to adopt, while simultaneously occluding the RNAP-binding regions (Figure 3). The Group 3 *Aquifex aeolicus* σ^FliA^/FlgM structure revealed that the σ^FliA^ σ_2_, σ_3_ and σ_4_ domains are organized as a compact unit with the extended helical FlgM wrapped around the outside, blocking the major RNAP binding determinants in σ_2_ and σ_4_ [64]. Recently, a new class of ECF anti-σ factors was proposed to act in a similar way [65]. The structure of the cobalt and nickel resistance regulator σ^CnrH^ from the β-proteobacterium *Cupriavidus metallidurans* in complex with the cytoplasmic domain of its anti-σ factor, CnrY, revealed that the σ_2_ and σ_4_ domains are embraced in a closed conformation by two CnrY helices, such that the σ_4_ domain is buried against the −10 interaction surface in σ_2_ and CnrY blocks the σ^CnrH^ RNAP-binding determinants [65] (Figure 3). Remarkably, despite no primary sequence similarity, this structure can be superimposed on that of a complex between the σ factor mimic PhyR and the anti-σ factor NepR from α-proteobacteria [66,67] suggesting that this might be a widespread mechanism.

## 5. Mechanisms for Triggering σ Factor Release from Anti-σ Factors

**Figure 4:**
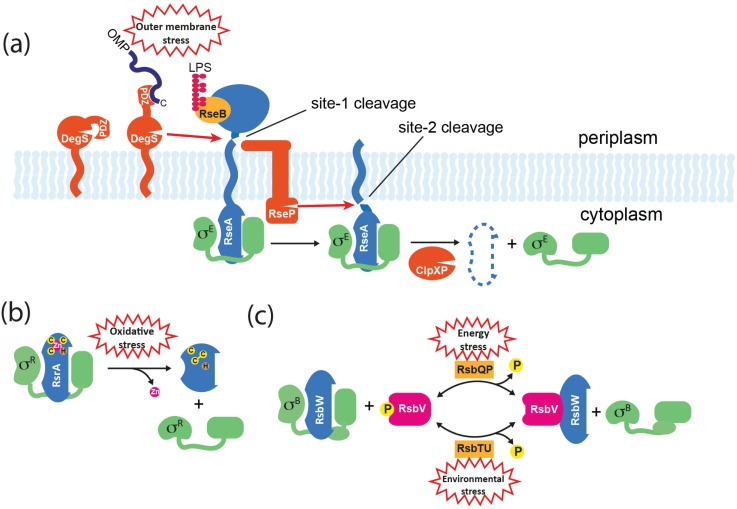
Mechanisms for σ factor release from anti-σ factors. (**a**) Activation of σ^E^ in *E. coli*. The membrane-spanning anti-σ RseA binds σ^E^ through its cytoplasmic ASD. The C-termini of OMPs that accumulate in the periplasm activate DegS protease through binding to its PDZ domain, resulting in site-1 cleavage of RseA. DegS–dependent cleavage of RseA is inhibited by RseB, and this can be relieved by the accumulation of lipopolysaccharide (LPS) in the periplasm that binds directly to RseB. Site-1 cleavage is sensed by RseP, which subsequently catalyzes site-2 cleavage of RseA on the cytoplasmic side of the membrane, releasing a soluble σ^E^/RseA complex. σ^E^ is released when the RseA ASD is finally degraded by ATP-dependent proteases such as ClpXP. (**b**) Activation of σ^R^ in *S. coelicolor.* RsrA binds to and inactivates σ^R^ via its ZASD domain. The RsrA zinc ion is coordinated by three cysteine (C) residues and one histidine (H). In response to oxidative stress, RsrA forms at least one disulphide bond, which concomitantly displaces the zinc and causes a structural change that prevents σ^R^ binding. The system can be reset by the reduction of RsrA by cellular thiol-disulphide oxidoreductases such as thioredoxin that are activated by σ^R^. (**c**) Activation of σ^B^ in *B. subtilis.* RsbW is an anti-σ factor/kinase that binds to σ^B^, and additionally inactivates its alternative binding partner RsbV by phosphorylating it to RsbV-P. Activation of σ^B^ occurs by partner-switching when RsbV-P is dephosphorylated by alternative phosphatases (RsbTU or RsbQP) in response to environmental or energy stresses, which allows RsbV to bind and sequester RsbW.

The mechanism for releasing cytoplasmically-located σ factors in response to signals that often stem from the external environment is understood in only a small number of cases. They can be broadly divided into partner-switching, direct sensing and regulated proteolysis mechanisms (Figure 4). In the case of partner-switching and regulated proteolysis, an emerging theme is the integration of distinct signals involving separate input pathways that enable σ activation in response to varied environmental and physiological cues.

### 5.1. Regulated Proteolysis

Regulated intramembrane proteolysis (RIP) is a mechanism for signal transduction across membranes that is found in all kingdoms [68] and is commonly used to control ECF σ factors in response to extracytoplasmic stimuli. It usually involves the sequential cleavage of a membrane-traversing anti-σ by an external “site-1” protease followed by cleavage in the plane of the membrane by a “site-2” protease, resulting in the release of the cytoplasmic domain. In *E. coli*, RIP controls the release of σ^E^ from RseA in response to outer membrane dysfunction (Figure 4a). Site-1 proteolysis of the C-terminal extracytoplasmic domain of RseA is catalyzed by the periplasmic protease DegS, following the accumulation of C-terminal peptides from unfolded outer membrane proteins (OMPs) that activate DegS by binding to its PDZ domain [69,70]. This initial cleavage generates a substrate for the site-2 protease RseP (YaeL) that cleaves RseA on the cytoplasmic side of the membrane, thereby releasing a σ^E^/RseA-ASD complex into the cytoplasm [71,72]. Although σ^E^ is still unable bind to RNAP at this point, the site-2 cleavage of RseA leaves a C-terminal sequence that directs its rapid degradation by the ClpXP system, thereby releasing σ^E^ [73]. However, to fully induce σ^E^ another regulator needs to be inactivated. RseB is a periplasmic protein that binds to RseA to inhibit DegS-dependent cleavage [74] (Figure 4a). Recently it was shown that inhibition by RseB is prevented by lipopolysaccharide intermediates that bind directly to RseB. Therefore the activation of σ^E^ requires two distinct stress signals (OMPs and LPS assembly components) with “AND” logic, which ensures that σ^E^ is not switched on inadvertently [75].

An analogous system occurs in *B. subtilis* where σ^W^ is sequestered by the trans-membrane ZASD anti-σ factor RsiW. In response to a signal that is generated by antimicrobial peptides or other agents that damage the cell envelope, RsiW is sequentially cleaved by the site-1 protease PrsW and the site-2 protease RasP [76,77]. Unlike DegS, PrsW is an integral membrane protease, consistent with the absence of a periplasm in this Gram-positive organism. Another difference is that the extracytoplasmic portion of the site-1 cleavage product needs to be trimmed before it becomes a substrate for RasP [78]. As is the case in *E. coli*, site-2 cleavage reveals a C-terminus that targets the protein for degradation by the ClpXP system, thereby fully releasing the σ [79].

Finally, RIP is involved in the activation of σ factors involved in iron-uptake in *P. aeruginosa* by cell surface signalling. In *P. aeruginosa* σ^FpvI^ and σ^PvdS^ direct expression of the ferripyoverdine siderophore outer membrane receptor (FpvA) and pyoverdine synthesis, respectively, and unusually are controlled by the same anti-σ factor, FrvR [80]. When pyoverdine binds to FpvA, a signal is transferred via the periplasmic domain of FpvA to FrvR, resulting in the degradation of FrvR by the RseP orthologue MucP [81]. As with *E. coli* σ^E^ and *B. subtilis* σ^W^, the remaining σ-bound portion of FrvR is thought to be degraded by the Clp protease system [81,82].

### 5.2. Direct Sensing

In some cases a signal is sensed directly by the anti-σ, causing a conformational change that releases the σ factor. The first-described example of this class was the σ^R^-RsrA system of *S. coelicolor* [83]. As mentioned above, RsrA is a member of the ZASD family but lacks membrane spanning or extracytoplasmic domains, and is cysteine-rich. In response to oxidative stress, RsrA forms an intramolecular disulphide bond between two cysteines including one that occurs in the ZASD motif [84]. This causes the expulsion of zinc and the stabilisation of a form of RsrA that is unable to interact with σ^R^ [84] (Figure 4b). The induction of the σ^R^ regulon is transient, partly through the recycling of RsrA following its reduction by members of the σ^R^ regulon such as thioredoxin [57,58,83]. It should be noted that most members of the ZASD family are unlikely to sense redox change, and instead use zinc in a purely structural role. However, other redox sensing anti-σ factors have been identified, including the integral membrane protein OsrA that modulates an oxidative stress response by σ^EcfF^ in *Bradyrhizobium jabonicum* [85]. Mutation of a single periplasmically-located cysteine residue removed the ability of OsrA to release σ^EcfF^ in response to oxidative stress, suggesting that the mechanism for σ activation involves the modification of this residue. Conformational changes in anti-σ factors that do not involve redox change have also been proposed. In *C. metallidurans* the binding of cobalt or nickel to the periplasmic domain of the sensor CnrX is thought to cause a conformational switch in the associated anti-σ CnrY, which then releases σ^CnrH^ [86]. Finally, in *B. subtilis* activation of σ^V^ in response to lysozyme treatment appears to occur via a combination of direct sensing and RIP mechanisms [87]. The anti-σ RsiV binds directly to lysozyme, which is thought to invoke an allosteric change that makes RsiV susceptible to site-1 proteolysis. Remarkably, the site-1 protease appears to be a signal peptidase responsible for general protein secretion, whereas site-2 proteolysis is catalysed by RasP, as is the case for RsiW (see above).

### 5.3. Partner-Switching

Partner-switching mechanisms are widespread, particularly among Gram-positive bacteria, and have been extensively studied for the Group 3 sigma factors σ^B^ and σ^F^ in *B. subtilis.* Although these σ factors use different sensing mechanisms to modulate their district biological activities, the fundamental components that control σ activity are homologous. There are four basic components (named for the σ^B^ system): σ factor (σ^B^); anti-σ factor/protein kinase (RsbW); anti-anti-σ factor (RsbV); and input phosphatase complex (RsbTU or RsbQP) (Figure 4c). In unstressed cells σ^B^ is held inactive by RsbW; RsbW can alternatively bind to the non-phosphorylated form of RsbV, but RsbW itself anatagonizes this interaction by phosphorylating RsbV to RsbV-P (Figure 4c). The phosphorylation status of RsbV is therefore crucial to the active state of σ^B^. To activate the general stress response, RsbTU or RsbQP dephosphorylate RsbV, thereby allowing RsbV to bind RsbW and liberate σ^B^. RsbTU and RsbQP are alternative phosphatases consisting of a phosphatase component (RsbU/RsbP) and a cognate activator (RsbT/RsbQ), that allow the integration of environmental and energy stress, respectively. Environmental stresses such as heat lead to the activation of RsbU phosphatase via a complex pathway that involves at least 10 proteins including a 1.8 MDa cytoplasmic protein complex known as the stressosome, which is the source of RsbT [46,88]. The activity of the RsbP phosphatase is thought to be modulated by the direct sensing of an unknown energy-related metabolite that is possibly provided by its partner hydrolase protein, RsbQ [89].

In α-proteobacteria, including epiphytes *Methylobacterium extorquens* and *Sphingomonas melonis*, and *Caulobacter cresentus*, an unusual partner-switching mechanism involving three often genetically-linked gene products exists to control the general stress response. Using the *M. extorquens* nomenclature, the key players are σ^EcfG1^, its anti-σ factor NepR and PhyR. PhyR was originally identified as an activator of stress-related genes associated with growth in the harsh environment of the leaf surface and unusually consists of an N-terminal ECF σ-like domain and a C-terminal response regulator receiver domain [90]. However, rather than acting as a σ, PhyR is a σ factor mimic, regulating transcription indirectly by binding to NepR when the receiver domain is phosphorylated, thereby freeing σ^EcfG1^ [91]. The σ-like domain of PhyR (PhyR_SL_) consists of σ_2_- and σ_4_-like subdomains and is occluded by the receiver domain in the unphosphorylated form [92]. This regulatory arrangement allows multiple stress signals to converge on PhyR, leading to the activation of σ^EcfG1^ and this is the case in *S. melonis* where seven histidine kinases were recently implicated in the phosphorylation of PhyR in response to distinct stress signals [93].

## 6. Indirect Regulation of Alternative σ Factors by Primary σ Factor Control

In *E. coli*, σ^70^ has a higher affinity for RNAP than alternative σ factors and is present in excess of RNAP that is not involved in elongation [94,95,96]. This generates a competitive environment and raises the question of how alternative σ factors can effectively access RNAP. Under conditions (e.g., nutrient starvation) where the expression of growth-related genes such as rRNA operons is rapidly reduced, the resulting increase in free core RNAP would be expected to lead to a passive rise of alternative holoenzyme forms [97]. An alternative mechanism is to sequester σ^70^, which appears to be the role of Rsd in *E. coli*. Rsd was discovered in a search for an anti-σ factor that might promote σ^S^ activity during stationary phase [98]. As is the case for other anti-σ factors, Rsd interacts with both σ^70^_2_ and σ^70^_4_ domains [99] with the latter shown to prevent binding both to the β-flap-tip-helix and to −35 promoter elements [100]. Interestingly, although there is only a two-fold increase in Rsd from exponential to stationary phase, Rsd associates effectively with σ^70^ only during stationary phase [95]. A possible explanation is that Rsd can alternatively bind the non-phosphorylated form of the phosphoenolpyruvate-dependent phosphotransferase system (PTS) control protein HPr, which is the dominant form of HPr when cells are growing exponentially with glucose as carbon source [101]. HPr therefore appears to act through a partner-switching type mechanism with Rsd to control levels of σ^70^ holoenzyme. Nonetheless, the importance of Rsd in *E. coli* remains enigmatic because null mutants have little discernable phenotype. Whereas Rsd was identified biochemically, its orthologue AlgQ in *Pseudomonas aeruginosa* was identified genetically as a positive regulator for alginate production, a key virulence factor that contributes to long term persistence of chronic lung infections in cystic fibrosis patients [102]. The key enzyme for alginate production is encoded by *algD*, which is transcribed by the ECF σ factor σ^AlgU^ [103]. The evolution of mucoid strains that overproduce alginate often occurs as a result of mutations in the downstream encoded anti-sigma factor MucA [104,105]. The expression of *algD* additionally relies on AlgQ (also known as AlgR2), which is 55% identical to Rsd and binds to σ^70^ in *Pseudomonas* spp. [106,107]. It has therefore been proposed that AlgQ activates σ^AlgU^ indirectly, by sequestering σ^70^ [107]. Consistent with such an indirect effect, AlgQ also affects the expression of pyoverdine uptake and production genes by σ^PvdS^ and σ^FpvI^. AlgQ mutants show reduced levels of pyoverdine, which can be suppressed by the over-expression of σ^PvdS^ or augmented by the over-expression of σ^70^ [108]. An interesting future question is whether HPr also plays a role in *Pseudomonas aeruginosa*, linking alginate, siderophore and other virulence gene expression to central metabolism.

## 7. Conclusions

Sigma factors are remarkable proteins that play both mechanistic and regulatory roles in transcription. Significant progress has uncovered many of the molecular details of σ action during initiation, although the vast majority of research has been conducted with primary σ factors. One future challenge is therefore to understand how the structural divergence of alternative σ factors translates to functional differences. Genome sequencing efforts have allowed us to gauge the diversity and distribution of σ factors with some striking examples of expansion. This is particularly the case in the ECF subfamily where there is enormous signaling diversity with many novel mechanisms of σ control and sensory perception yet to be uncovered [109]. Primary sequence analysis is providing tantalizing clues with some ECF σ factor groups containing unusually large C-terminal extensions with possible regulatory or localization roles, and others showing microsynteny with post-translational modification proteins such as serine/threonine protein kinases [54]. We still have a poor understanding of the extent and biological importance of cross-talk between σ-based signaling pathways and how bacteria manage to insulate transcriptional responses when many σ factors are present. Furthermore, although progress has been made in the γ-proteobacteria, we need to further understand how alternative σ factors function in the face of stiff competition from primary σ factors and the biological relevance of passive control of alternative σ factors through changes in the availability of RNAP. Finally, understanding the diverse roles and regulation of σ factors will not only help us to understand how bacteria sense and respond to environmental change, but also provide opportunities for the development of synthetic devices for genetic control. σ factors have several attractive features for this purpose, including modularity that allows the development of chimeric proteins with novel promoter-recognition characteristics, control by anti-σ factors allowing their incorporation into genetic switches, and the ability to function in diverse organisms on account of the conservation of RNAP [56].

## Abbreviations

ASD: Anti-sigma domain
σR: Sigma region
ZASD: Zinc binding anti-sigma domain
RIP: Regulated intramembrane proteolysis
RNAP: RNA polymerase
RP: RNA polymerase/ promoter complex
NCR: Non-conserved region of sigma
ECF: Extracytoplasmic function
OMP: Outer membrane protein
LPS: Lipopolysaccharide
